# Dental Erosion from an Excess of Vitamin C

**DOI:** 10.1155/2014/485387

**Published:** 2014-08-04

**Authors:** Priya Bahal, Serpil Djemal

**Affiliations:** Restorative Dentistry, King's College Dental Institute, Camberwell, London SE5 9RS, UK

## Abstract

Acid erosion of enamel is the chemical dissolution of the superficial layers of teeth without the presence of bacteria. If the presence and exposure of a demineralising agent such as vitamin C is frequent and prolonged, it can lead to significant tooth wear. This case report discusses one such presentation and as a result of the occlusal relationship, this serves to effectively demonstrate the localised effects of vitamin C-induced acid erosion. The management of localised tooth wear with composite restorations utilising the Dahl principle to replace lost tooth tissue is also reported. *Clinical Relevance*. Patients should be made aware of the erosive nature of chewable vitamin C tablets and their potentially harmful effects on the dentition if consumed in excess. *Objective Statement*. The reader should understand the clinical implications of an excessive intake of vitamin C. This demonstrates the importance of the manufacturer's instructions and the clinician's role in advising patients with regard to the correct therapeutic doses.

## 1. Introduction

Dental erosion can be described as the irreversible loss of tooth structure due to the chemical process of acid dissolution which does not involve plaque bacteria. Sources of erosion can be intrinsic such as acid reflux and vomiting or extrinsic, from the ingestion of food, drink, or medication. Lifestyle and occupations can also influence the multifactorial pattern of tooth wear, and erosion frequently coexists with attrition and/or abrasion. The degree to which each of these factors contributes to the overall clinical picture will vary from patient to patient, and it is important that a detailed medical and clinical history is taken with patient-specific questioning in order to form a comprehensive differential diagnosis.

Vitamin, derived from the word* vitamine*, describes a group of organic compounds which are essential to life. They are present in natural products or made synthetically and are required in small quantities in the diet of animals and humans. Many vitamins are synthesised within the human body; however, some, such as vitamin C, cannot be produced naturally and so must be gained from sources within the diet.

Vitamin C (ascorbic acid) is a water-soluble compound and is necessary for enzyme activation, oxidative stress reduction, and immune function. It is present in fruit and vegetables including oranges, peppers, and broccoli. Chewable vitamin C tablets have been reported to have a pH of 2.3 and the critical point at which enamel dissolves is around pH 5.5 [1]. The buffering potential of saliva and the salivary pellicle act to counteract the effects of the acidic challenge. If, however, the protective potential is limited and the exposure of acid is continual and at a lower than critical pH, the irreversible loss of dental hard tissues will ensue.

On a microscopic level, the pattern of erosion initially follows the prismatic structure of enamel where either the prism cores or interprismatic areas dissolve first. This dissolution then leads to a less regular pattern within the aprismatic enamel and finally, once the enamel is breached, this affects the peritubular and then intertubular dentine [2]. Clinically, this can be seen as hard, smooth surfaces with cupping of the occlusal surfaces. If the process of wear is slow, the dentine is frequently stained and often, when this develops as a slow, chronic process, the patient will not experience any dentine sensitivity despite the significant tooth wear.

This report describes an extreme example whereby chewable vitamin C tablets were consumed on a frequent basis over a three-year period leading to marked tooth surface loss. This case illustrates how dental erosion, if not controlled, can lead to potentially harmful effects on both the dentition and stomatognathic system.

## 2. Case History 

**Figure 1:**
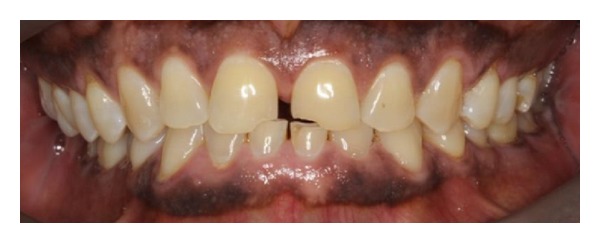
Appearance at presentation.

A 51-year-old fit and healthy Nigerian male engineer presented himself to the emergency department of King's College Dental Institute complaining of a left-sided jaw ache, an “uneven bite,” and being unhappy with the appearance of his front teeth (Figure 1). He explained that this pain had been ongoing for the previous two to three years; however, this “ache” had become more apparent over the previous two months. He explained that the issues of appearance had been raised following comments from family members.

Extraoral examination revealed slight tenderness to palpation on the left masseter and lateral pterygoid muscles. He exhibited good opening with no deviation or marked antegonial notching.

**Figure 2:**
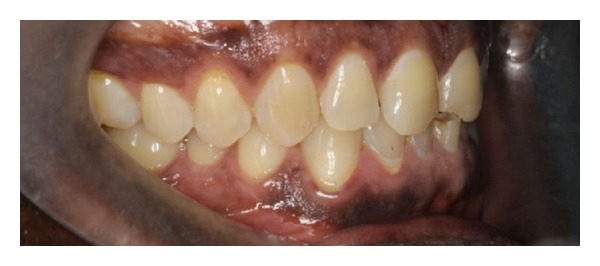
Photograph of the scissors bite. Note the reduction in height of the lower anterior teeth.

Intraoral examination revealed healthy soft tissues with racial pigmentation, an unrestored dentition, and fair oral hygiene. The periodontal status appeared stable with no tooth mobility or bleeding on probing. The patient had a class one incisal relationship with a right sided scissors bite. (Figure 2), and the UR6 and UR7 were partially overerupted.

**Figure 3:**
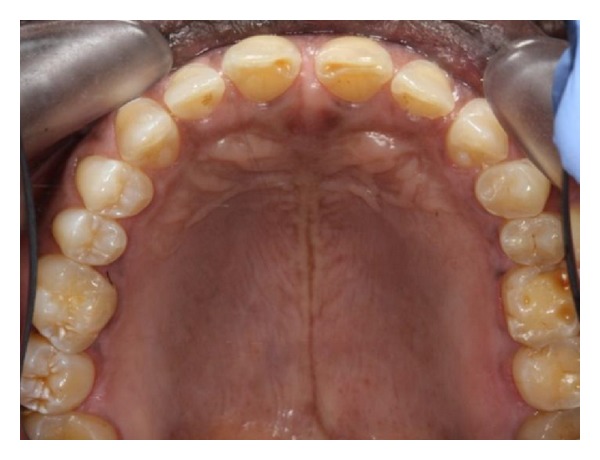
Marked tooth surface loss affecting the upper incisors and left posterior teeth only.

Marked tooth wear was evident on the upper and lower incisors and left-sided posterior teeth only (Figure 3). There was loss of the outer two-thirds of enamel on the mesial aspects of the upper 5 incisors and the lower incisors had lost around a third of their clinical crown height. Significant tooth wear was present to varying degrees on the occlusal aspects of the left premolar and molar teeth only; all teeth on the right side were of normal anatomy and form. Chipping of the lower lateral incisors was noted and there was characteristic “cupping” of the enamel with staining of the underlying dentine.

**Figure 5:**
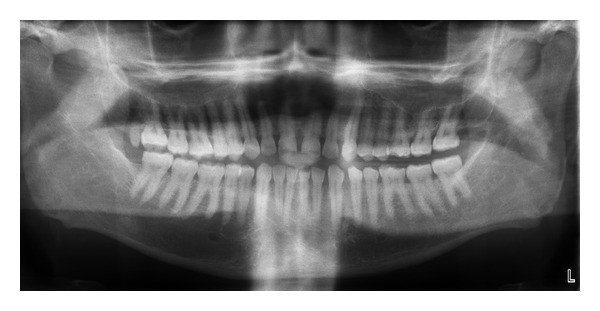
Full mouth OPT.

A full mouth OPT (Figure 5) was taken to assess any condylar abnormalities and to exclude any dental pathology. Radiographic examination revealed around 5% generalised horizontal bone loss and a localised vertical defect was associated with the UL6 and UL7. A supernumerary was present distal to the UR8 and the occlusal anatomy of the left-sided molars appeared significantly flatter in comparison to their contralateral teeth.

A detailed history was taken to establish the cause of the localised tooth surface loss. The patient was questioned to assess if any specific intrinsic or extrinsic factors could have contributed to the patterns observed. The patient admitted to a three-year history of chewing multiple vitamin C tablets on almost a daily basis.

The patient explained that he had been advised by a friend to increase his intake of vitamin C as it was beneficial for general health. He felt that the tablet form would be a convenient source of the vitamin and so he purchased “Superdrug” (Croydon, United Kingdom) own-brand chewable vitamin C tablets from various Superdrug stores. 5-6 of these chewable tablets would be kept in his pocket each day thereby enabling him to continuously suck and chew on these each weekday during his 90-minute morning commute to work. In addition, the patient developed a habit to “snack” on these tablets throughout the morning leading to lunchtime when he would then eat his first meal of the day. This continual intake of tablets meant that there would be no more than a 45–50-minute break between each tablet being consumed. He explained that, during the winter months, the quantity and frequency of the tablets would increase as a result of the colder weather. He reported no systemic adverse effects.

The following diagnoses were made:

1. left-sided myofacial pain,
2. erosive and abrasive tooth surface loss,
3. right-sided scissors bite.

The oral and maxillofacial team supplied the patient with a lower soft acrylic mouth guard in an attempt to ease the left-sided facial pain. Following a three-month review and relief of his symptoms, the patient was referred to the joint orthodontic-restorative team to assess if any treatment was indicated for correction of the crossbite and to see if this would be implicated with any potential restorative treatment for the tooth surface loss.

At the time of presentation to the joint orthodontic-restorative clinic, the patient's complaints were limited to the aesthetic issues only. He no longer had pain but still wished to proceed with treatment to aid his appearance.

The following treatment options were discussed:

(1) fixed orthodontics to correct the scissors bite,
(2) direct composite build-ups on

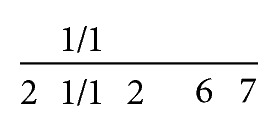
(1)
 utilising the Dahl concept to create interocclusal space.

The erosive nature of vitamin C was explained and detailed advice was given with regard to its appropriate intake. As the primary source of the erosion and abrasion was no longer present in the patient's diet, the treatment was limited to direct, freehand composite build-ups of the anterior teeth and the LL6 and LL7.

**Figure 6:**
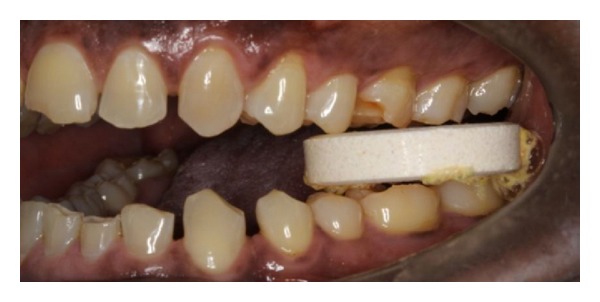
Posterior teeth being used to grind and chew the tablets.

The teeth were initially cleaned with pumice and water, etched with 37% phosphoric acid and then restored with direct composite resin (Gradia, Belgium) shades B2 and B3 to replace the lost tooth tissue (Figure 6). Posteriorly, the composite was bought up to the enamel rim and, anteriorly, the restorations were placed again freehand with no bevelling of the enamel.

At a three-month review the patient had no complaints and the composite restorations were sound. He was happy with both the appearance and function of the restorations and had adapted well to the slight increase in occlusal vertical dimension. He continued to be in good general health and was obtaining his daily vitamin C intake from fruit and vegetables.

## 3. Discussion 

It is well known that vitamin C (ascorbic acid) has a low pH and so can contribute to dental erosion. In comparison to citric and phosphoric acid, which is found in many carbonated drinks, ascorbic acid has been proven to be relatively more erosive. The erosive potential of vitamin C and that in particular which was caused by the long-term use of chewable vitamin C tablets was clearly illustrated in 1982 [3]. Therefore, over thirty years ago this issue was highlighted and it was suggested that, as professionals, we should help our patients through supportive education and advice to focus on the causation and prevention of dental erosion throughout their lives. This case however has demonstrated that despite our increased awareness and knowledge of the topic, patients can still remain oblivious to the harm that an excess of vitamin C can cause on the dentition.

As vitamin C cannot be stored in the body, it is imperative that some is obtained from the diet on a daily basis to maintain health. The exact amount required for an individual is dependent upon their gender, weight, and general health but it has been suggested that, for an average 70 kg male, around 60 mg/d of vitamin C is required on a daily basis [4]; this can be gained from one large orange.

One study has shown that individuals who consume less than 50 mg of vitamin C daily have twice the risk of developing cancer than one who consumes more than 100 mg [5]. It should be noted that once an individual's daily requirement has been reached, any additional vitamin C becomes surplus to the body and is excreted as waste in urine. With regard to safety, there have been reports on individuals taking doses of up to 1,000 mg per day for over one year with no adverse effects on their general health. This is of course separate from any adverse effect that could occur on the dental hard tissues.

It has been shown that patients commonly misunderstand dosage information on prescription drug labels [6], and it can be argued that over-the-counter supplementary products require an increased level of knowledge to ensure compliance is within the safe range. Supplementary medicaments are not always directly prescribed by a medical practitioner and so there is arguably more scope for misinterpretation of drug usage. Often as was the case here, patients are drawn towards increasing their vitamin C intake for so-called “health benefits.”

Systemically, it has been reported that vitamin C can be beneficial in the prevention of heart disease and certain cancers due to its antiartherosclerotic and anticariogenic properties [7]. It can be seen how the general public can be guided towards seeking additional sources of vitamin C with a view to improve their general health following health promotion.

The degree of acid erosion experienced by an individual will depend on numerous factors, for example, the titratable acidity of the tablet, the pH of the individual's saliva, and of course their own frequency and pattern of use. It has been shown that tablets containing 60 mg, 250 mg, or 500 mg of ascorbic acid will all cause a reduction in salivary pH. Furthermore a tablet containing 500 mg of vitamin C, a so-called “megadose,” will not only dramatically cause a pH drop to below the critical pH, but also cause a sustained drop below the pH 5.5 for up to 25 minutes after the initial acidic challenge [8]. This illustrates the highly erosive potential of the tablets and in this case the tooth surface loss was exacerbated from the continual direct contact with the teeth. The acid served to initially soften the surface enamel making it more prone to wear from the abrasive tablets.

**Figure 7:**
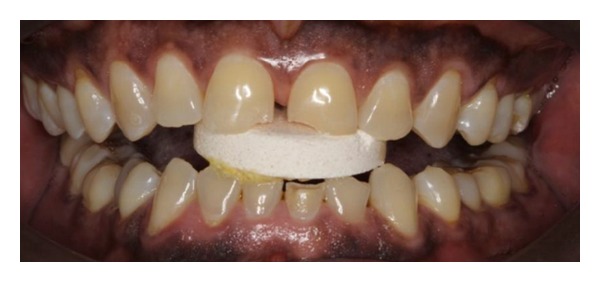
Anterior teeth being used to hold tablet horizontally between occluding teeth.

**Figure 8:**
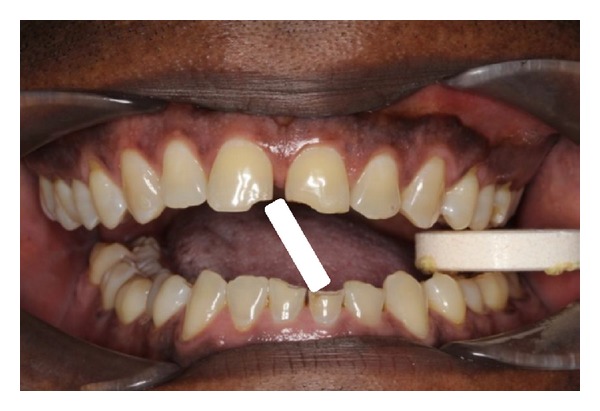
Possible anterior positioning of tablets superimposed onto photograph.

When used in moderation and in line with the manufacturer's instructions, chewable vitamin C tablets pose no obvious risks to health or the dentition. This case has shown that if their use is excessive, frequent, and limited to certain pairs of occluding teeth for a prolonged time, it will lead to significant tooth surface loss. Here the source of acid was via large, hard, round tablets and so there was limited physical space available for the saliva to act as a buffer during episodes of acid attack. The teeth which were spared from the acidic challenge showed no evidence of erosion which demonstrates that otherwise the patient had normal saliva which was capable of acting as a suitable buffer under normal physiological conditions. The presence of a scissors bite caused the patient's chewing habit to be limited to the anterior and left posterior teeth only (Figures 7 and 8). This corresponds with the clinical result as the molar teeth, those which would have been used for chewing and grinding, showed significant areas of erosion on the occlusal aspects. The left-sided occlusal surfaces had characteristic shallow, cupped surfaces which were hard with stained dentine which was non-carious and indicative of an extrinsic source of acid. Also, as a result of their increased surface area, the molar teeth had more tooth surface loss than the premolar teeth on the same side. The canine had only normal physiological tooth wear which was consistent with the patient's age.

**Figure 9:**
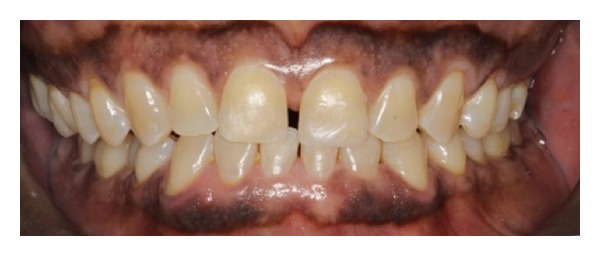
Post-op direct composite build ups.

It has been shown that extrinsic acid erosion was due to the ingestion of carbonated and sports drinks and, more recently, so-called smoothies which are all popular choices with the younger generation and this has resulted in an increased prevalence of acid erosion in this cohort. Typical presentations are thinning of the labial/facial aspects of enamel on the upper teeth with concave-shaped lesions. With intrinsic sources such as vomiting or reflux, the palatal surfaces of the upper teeth are usually affected, leaving the labial and occlusal surfaces of the upper teeth and the lower teeth untouched. The observed pattern of tooth surface loss again correlated with the patient's history as he reported initially holding each tablet vertically (Figure 9) between the anterior teeth before moving it to the left posterior region for continual sucking and biting.

In all patients who suffer from erosive tooth surface loss, the initial priorities are to establish its cause and to consequently prevent further irreversible loss of tooth structure. This could be through patient awareness and dietary modification or it could require further investigation to detect an underlying cause of intrinsic acid. In this case, the patient had ceased chewing the vitamin C tablets and replaced this habit with a moderate intake of fruit and vegetables.

The pragmatic, conservative approach to treatment of the tooth wear was both cost-effective and acceptable to the patient. The material was placed in compression in an attempt to increase its longevity, and being fully dentate and male, the patient coped very well with the slight increase in occlusal vertical dimension in line with the Dahl concept [9]. Following the treatment and a period of adaptation, a balanced occlusion was reestablished following relative intrusion of the anterior teeth and extrusion of the posteriors.

## 4. Summary

A patient demonstrated moderate to severe dental erosion which was attributed to the almost daily intake of chewable vitamin C tablets over the course of three years. Once the cause was established, detailed preventative advice was given and localised composite resin restorations were placed to replace the lost tooth tissue. The patient coped well with the treatment and his vitamin C intake was reduced to a moderate, healthy intake. This case demonstrates how, as clinicians, we are responsible for advising the patients on potential sources of acid erosion and guiding them towards safe levels of use to prevent any adverse effects on their dentition.

## Figures and Tables

**Figure 4:**
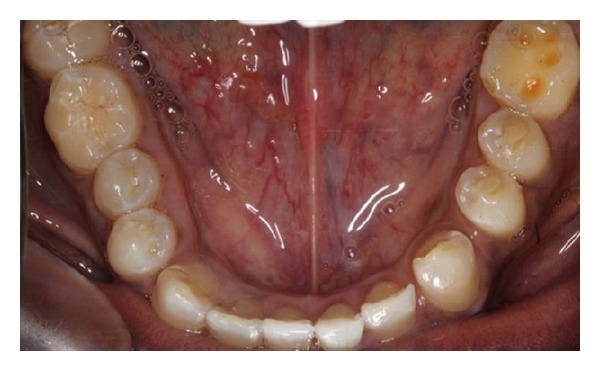
Tooth surface loss affecting teeth in the lower left quadrant. Note the normal anatomy of the teeth on the right side in Figures 3 and 4.

## References

[1] Blacker SM, Chadwick RG (2013). An in vitro investigation of the erosive potential of smoothies. *The British Dental Journal*.

[2] Meurman JH, Ten Cate JM (1996). Pathogenesis and modifying factors of dental erosion. *European Journal of Oral Sciences*.

[3] Giunta JL (1983). Dental erosion resulting from chewable vitamin C tablets. *Journal of the American Dental Association*.

[4] Levine M, Conry-Cantilena C, Wang Y (1996). Vitamin C pharmacokinetics in healthy volunteers: evidence for a recommended dietary allowance. *Proceedings of the National Academy of Sciences of the United States of America*.

[5] Block G (1991). Vitamin C and cancer prevention: the epidemiologic evidence. *The American Journal of Clinical Nutrition*.

[6] Wolf MS, Davis TC, Shrank W (2007). To err is human: patient misinterpretations of prescription drug label instructions. *Patient Education and Counseling*.

[7] Naidu KA (2003). Vitamin C in human health and disease is still a mystery? An overview. *Nutrition Journal*.

[8] Hays GL, Bullock Q, Lazzari EP, Puente ES (1992). Salivary pH while dissolving vitamin C-containing tablets. *The American Journal of Dentistry*.

[9] Poyser NJ, Porter RWJ, Briggs PFA, Chana HS, Kelleher MGD (2005). The Dahl concept: past, present and future. *The British Dental Journal*.

